# PLAST: parallel local alignment search tool for database comparison

**DOI:** 10.1186/1471-2105-10-329

**Published:** 2009-10-12

**Authors:** Van Hoa Nguyen, Dominique Lavenier

**Affiliations:** 1 Symbiose team-project, INRIA/IRISA, Campus de Beaulieu, 35042 Rennes Cedex, France; 2 ENS Cachan Bretagne, Campus de Ker Lann, 35170 Bruz, France

## Abstract

**Background:**

Sequence similarity searching is an important and challenging task in molecular biology and next-generation sequencing should further strengthen the need for faster algorithms to process such vast amounts of data. At the same time, the internal architecture of current microprocessors is tending towards more parallelism, leading to the use of chips with two, four and more cores integrated on the same die. The main purpose of this work was to design an effective algorithm to fit with the parallel capabilities of modern microprocessors.

**Results:**

A parallel algorithm for comparing large genomic banks and targeting middle-range computers has been developed and implemented in PLAST software. The algorithm exploits two key parallel features of existing and future microprocessors: the SIMD programming model (SSE instruction set) and the multithreading concept (multicore). Compared to multithreaded BLAST software, tests performed on an 8-processor server have shown speedup ranging from 3 to 6 with a similar level of accuracy.

**Conclusion:**

A parallel algorithmic approach driven by the knowledge of the internal microprocessor architecture allows significant speedup to be obtained while preserving standard sensitivity for similarity search problems.

## Background

Genomic sequence comparison is a central task in computational biology for identifying closely related protein or DNA sequences. Similarities between sequences are commonly used, for instance, to identify functionality of new genes or to annotate new genomes. Algorithms designed to identify such similarities have long been available and still represent an active research domain, since this task remains critical for many bioinformatics studies.

Two avenues of research are generally explored to improve these algorithms, depending on the target application. The first aims to increase sensitivity, while the second seeks to minimize computation time. With next generation sequencing technology, the challenge is not only to develop new algorithms capable of managing large amounts of sequences, but also to imagine new methods for processing this mass of data as quickly as possible [1].

The well-known Smith-Waterman (SW) algorithm, developed in 1981, is one of the first proposals to detect local similarities [2]. It uses a dynamic programming technique and has a quadratic complexity with respect to sequence length. A great effort has been made to obtain fast implementation on specialized hardware. Rognes [3] and Farrar [4] exploited the fine-grained parallelism of SIMD technology. Their implementations are respectively up to 6 and 13 times faster than the SSEARCH implementation [5]. More recent works use SIMD coprocessors, such as Graphics Processing Units (GPU) [6] or the CELL Broadband Engine [7]. Despite various attempts to accelerate the SW algorithm, its long computation time remains a major drawback. To increase speed, programs based on powerful heuristic methods, such as FASTA [5] or BLAST [8] have been developed. These greatly reduce execution time while maintaining a high level of sensitivity. Again, hardware coprocessors have been proposed to speed up these programs. These mostly use FPGA chips such as the SeqCruncher accelerator [9], the Mercury BLASTP implementation [10], the FPGA/FLASH board [11] or the specific FPGA-based BLAST platforms proposed in [12]. Implementation on the Cell Broadband Engine has also been experimented to make good use of the fine-grained parallelism of the BLASTP program [13].

The PLAST program is a pure software implementation designed to exploit the internal parallel features of modern microprocessors. The sequence comparison algorithm has been structured to group together the most time consuming parts inside small critical sections that have good properties for parallelism. The resulting code is both well-suited for fine-grained (SIMD programming model) and medium-grained parallelization (multithreaded programming model). The first level of parallelism is supported by SSE instructions. The second is exploited with the multicore architecture of the microprocessors.

PLAST has been primarily designed to compare large protein or DNA banks. Unlike BLAST, it is not optimized to perform large database scanning. It is intended more for use in intensive comparison processes such as bioinformatics workflows, for example, to annotate new sequenced genomes. Different versions have been developed based on the BLAST family model: PLASTP for comparing two protein banks, TPLASTN for comparing one protein bank with one translated DNA bank (or genome) and PLASTX for comparing one translated DNA bank with one protein bank. The input format is the well-known FASTA format. No pre-processing (such as formatdb) is required.

Like BLAST, the PLAST algorithm detects alignment using a seed heuristic method, but does so in a slightly different way. Consequently, it does not provide the same alignments, especially when there is little similarity between two sequences: some alignments are found by PLAST and not by BLAST, others are found by BLAST and not by PLAST. Nonetheless, comparable selectivity and sensitivity were measured using ROC curve, coverage versus error plot, and missed alignments.

Compared to BLAST (with its multithreading option activated), a speedup ranging from 3 to 6 can be obtained, depending on the amount and nature of the data to be processed. Furthermore, PLAST provides the best performance when large databases are involved.

## Implementation

PLAST implements a three-step, seed-based algorithm: (1) indexing, (2) ungapped extension and (3) gapped extension. An overview of the PLAST algorithm is presented below, followed by a more detailed description of the three steps.

### Overview of the PLAST algorithm

Like BLAST, the PLAST algorithm is based on a seed-based heuristic to detect similarities between two protein sequences. This heuristic supposes that two proteins sharing sufficient similarities include at least one identical common word of W amino acids. Then, from this specific word, larger similarities can be found by extending the search on the left and right hand sides. These words are called seeds because they are the starting point of the search alignment procedure.

The first step of the PLAST algorithm is to index the two protein banks using the subset seed concept [14]. Two tables of *T *entries are constructed, where *T *is the number of all possible subset seed keys. Each key entry is associated with a list of positions corresponding to all the occurrences of this subset seed in the bank.

The second step computes all the possible seed extensions. For each seed key, the two entries of the two tables are considered and each position of one list is compared with all the positions of the other list. In this context, comparing means computing small ungapped alignments by extending the subset seed on both sides.

The third step computes alignments including the gap penalty. This step is only triggered if the previous step has detected significant local similarity.

Based on these three steps, the principle of the PLAST algorithm can be described (sequentially) as follows:

Algorithm 1

1: *IT*_0 _← Index (bank-0)

2: *IT*_1 _← Index (bank-1)

3: for all possible seed key k

4:    *IL*_0 _← *IT*_0_[*k*]

5:    *IL*_1 _← *IT*_1_[*k*]

6:    for all elements i in *IL*_0_

7:       for all elements j in *IL*_1_

8:          if ungapped_extension (*IL*_0_[*i*], *IL*_1_[*j*])

9:             then gapped_extension (*IL*_0_[*i*], *IL*_1_[*j*])

Actually, this algorithm has great parallelism potential, since the computations of the 3 for all nested loops are independent. Basically, each seed extension can be performed in parallel. Thus, this implementation considers a first level of parallelism, called medium-grained parallelism, which is geared to multicore architectures and based on the multithreaded programming model. P threads corresponding to P available physical cores have the task of computing seed extensions simultaneously. This scheme corresponds to the parallelization of the outer for all loop (line 3). The algorithm is split into P+1 threads as given in Algorithm 2.

**Algorithm 2 **PLAST algorithm

Main thread

1: *IT*_0 _← Index (bank-0)

2: *IT*_1 _← Index (bank-1)

3: create P extension threads

4: K = 0

5: wait until K >= T

6:    merge thread results

P extension threads

1: while (K<T)

2: k = K++

3: *IL*_0 _← *IT*_0 _[*k*]

4: *IL*_1 _← *IT*_1 _[*k*]

5:    for all elements i in *IL*_0_

6:       for all elements j in *IL*_1_

7:          if ungapped_extension (*IL*_0_[*i*], *IL*_1_[*j*])

8:             then gapped_extension (*IL*_0_[*i*], *IL*_1_[*j*])

First, the main thread constructs two indexes before creating P extension threads. It sets a shared variable K to 0 (line 4), representing the key of the first subset seed value, and waits until all subset seed values have been processed. The extension threads increase K (line 2) and compute the extension related to K. The instruction k = K++ is atomic in order to prevent two threads from having the same K value. The last action of the main thread is to merge the results provided by each extension thread.

A second level of parallelism, called fine-grained parallelism, can be found in the two nested for all loops (lines 5 and 6, extension threads). Again, each seed extension between all the positions of the two index lists can be carried out simultaneously. Furthermore, this computation is very *regular *in that a score is systematically computed in the seed neighborhood. The value of this score indicates whether the alignments are significant or not. This regular computation is done using the SSE instruction set (Streaming SIMD Extensions) now available on all microprocessors. In this implementation, it allows the processor to calculate 16 scores in parallel.

Each step is now described in more detail.

### Step 1: bank indexing

**Figure 1:**
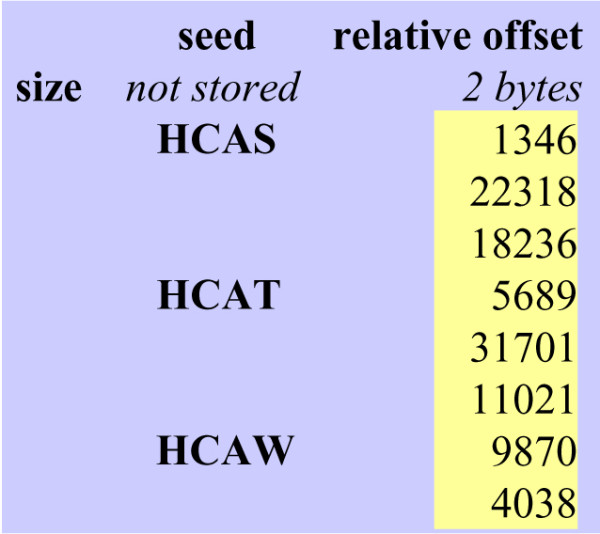
**Bank indexing**. Fragment of indexing scheme. For each seed key, a list of relative occurrence positions is stored on short integers.

Each protein bank is indexed using the same data structure as that shown in Figure 1. A list is made of all the positions in the protein bank of each seed key. A relative position, computed as the difference between two successive positions, is stored to minimize index size. As a result, the difference can be stored on a short integer (two bytes), rather than as an absolute position on a standard 4-byte integer. For infrequent subset seeds, however, the difference may exceed the dynamic range of short integers (2^16^). To circumvent this problem, false positive subset seed occurrences are added between two distance positions. The overhead introduced by these extra occurrences increases the size of the list by about 2%.

A subset seed is a word of W characters built by grouping together some amino acids [14]. The following 4-character subset seed structure can be considered as an example:

• character 1: A,C,D,E,F,G,H,I,K,L,M,N,P,Q,R,S,T,V,W,Y

• character 2: c = {C,F,Y,W,M,L,I,V}, g = {G,P,A,T,S,N,H,Q,E,D,R,K}

• character 3: A,C,f = {F,Y,W},G,i = {I,V},m = {M,L},n = {N,H},P,q = {Q,E,D},r = {R,K},t = {T,S}

• character 4: A,C,D,E,F,G,H,I,K,L,M,N,P,Q,R,S,T,V,W,Y

Here, the second character of a subset seed is either c or g. For example, the subset seed AcGL represents the words ACGL, AFGL, AYGL, AWGL, AMGL, ALGL, AIGL and AVGL in the 20 amino acid alphabet.

Whereas the BLAST algorithm requires two neighboring seeds of 3 amino acids to start the computation of an alignment, only one subset seed of 4 characters is used here. This offers the advantage of greatly simplifying computation by eliminating data dependencies and making it much more suitable for parallelism. An extension starts as soon as two identical subset seeds are found in two different protein sequences, thereby avoiding the need for any extra computation for managing pairs of seeds. In [14], it is shown that this subset seed structure and the BLAST approach exhibit comparable sensitivity.

PLAST requires 4 × 20^*W *^+ 2.02 × *n *bytes to index one sequence database, where W is the size of the subset seed being used (usually from 3 to 4) and *n *is the number of amino acids in the sequence database. To allow comparison of very large databases, PLAST automatically splits them into smaller fragments, which fit with the processor memory. Hence, databases of any size can be processed without further pre-processing.

### Step 2: Ungapped extension

As stated earlier, BLAST ungapped extension is run when two close seeds are detected. The extension starts from one seed and extends in both directions. The extension terminates when a running score falls below a threshold value. This technique allows BLAST to limit search space efficiently. As the size of the extension regions can vary from one sequence to another, however, this technique is not suitable for regular computation targeting SSE instructions.

**Figure 2:**
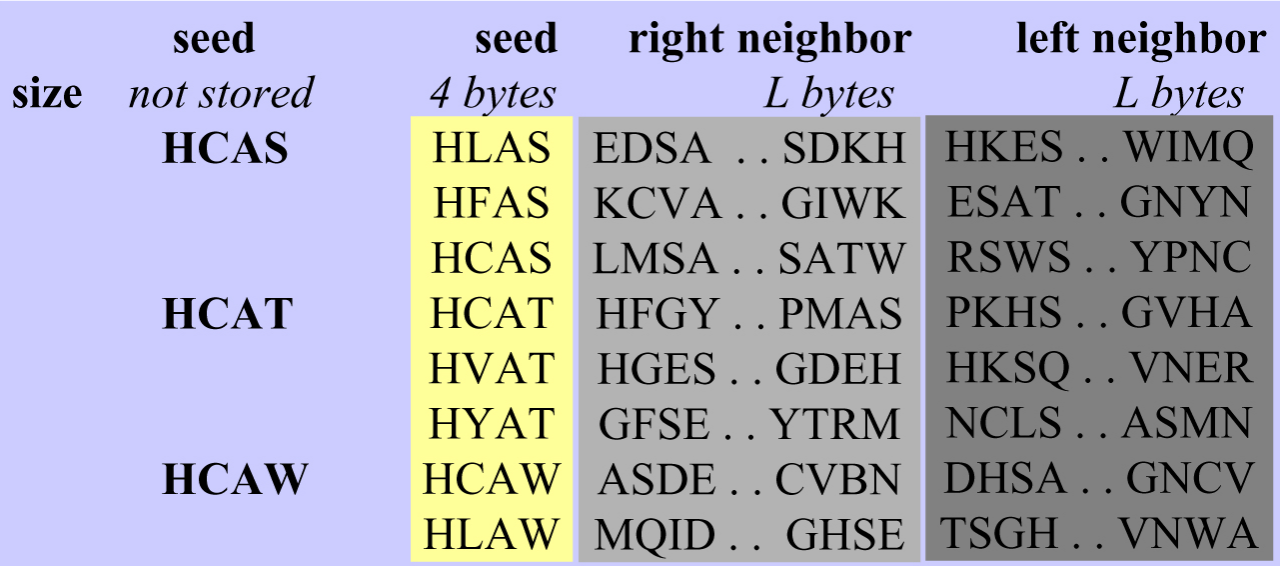
**Subsequence block**. Fragment of subsequence block. For each seed key, a list of subsequences is constructed. Each subsequence contains a seed and its right and left neighborhood.

The approach adopted here is different, performing an extension on a predefined size L, both on the left and on the right hand sides of the subset seed. More precisely, for a seed key k in the two index tables, *IL*0 has *K*_0 _elements and *IL*1 has *K*_1 _elements, meaning that *K*_0 _× *K*_1 _extensions must be processed. Thus, two blocks of subsequences *BLK*0_*k *_and *BLK*1_*k *_are constructed. Each subsequence is composed of a seed of W characters with its right and left extensions of L characters, as illustrated in Figure 2. Based on this data structure, the ungapped extension procedure between the *i*^*th *^subsequence of *BLK*0_*k *_and the *j*^*th *^subsequence of *BLK*1_*k *_is given in Algorithm 3.

The aim of this procedure is to compute a score related to the similarity between two protein subsequences of length (*W *+ 2 × *L*). It is split into two phases. The first computes a score by extending the right neighborhood (line 4-7). The maximal value is computed and set as the initial score for the left extension (line 9-12). At the end, the maximal score is compared to a predefined threshold value *τ*. If it is greater than *τ*, the couple of subsequences (*BLK*0_*k*_[*i*], *BLK*1_*k*_[*j*]) is a candidate for further processing (gapped extension).

**Algorithm 3 **ungapped extension procedure

1: *S*_0 _← *BLK*0_*k*_[*i*]

2: *S*_1 _← *BLK*1_*k*_[*j*]

3: *score *← 0; *max_score *← 0

4: for *x *= 1 to *W *+ *L*

5:    *score *← *score*+ Sub (*S*_0_[*x*], *S*_1_[*x*])

6:    if *score *> *max_score *then *max_score *← *score *endif

7: endfor

8: *score *← *max_score*

9: for *x *= *W *+ *L *+ 1 to 2 × *L *+ *W*

10:    *score *← *score*+ Sub (*S*_0_[*x*], *S*_1_[*x*])

11:    if *score *> *max_score *then *max_score *← *score *endif

12: endfor

13: if *max_score *≥ *τ *then return true endif

14: return false

Remember that for a specific seed key k, there are *K*_0 _× *K*_1 _extensions to process, and that all extensions can be computed in parallel (no data dependencies between these *K*_0 _× *K*_1 _processes). Hence, SSE instructions can be advantageously used to parallelize this procedure. The idea is to compute N scores in parallel using a SIMD scheme. In this processing mode, a score fits into 1 or 2 bytes and the SIMD register of the microprocessor simultaneously contains N scores. The extension procedure can thus be run in parallel between N subsequences of *BLK*0_*k *_and one subsequence of *BLK*1_*k*_.

In the implementation considered here, 16 scores are simultaneously computed on a 128-bit-wide register, forcing the score to fit between 0 and 255 (8 bits). As the score is computed on short subsequences, it rarely overflows. However, SSE instructions support saturating arithmetic on 8-bit unsigned values. Thus, if the result of an operation becomes greater than 255, it is automatically adjusted to 255.

**Figure 3:**
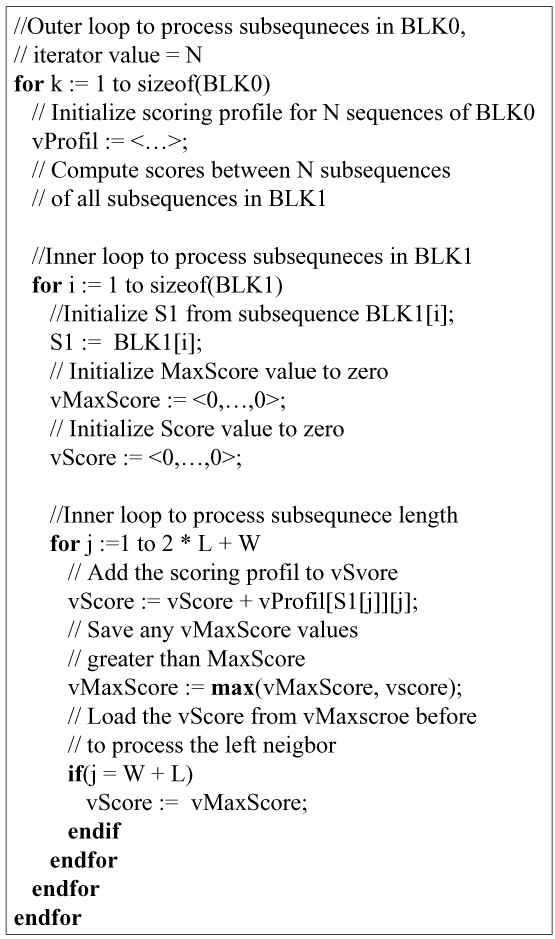
**pseudocode for *ungapped extension***. The pseudocode of ungapped extension procedure for 2 blocks of subsequences. Sixteen extensions are simultaneously processed and a score is stored on an 8-bit unsigned byte integer.

The last point that needs to be considered is how to manage negative scores. Owing to the limited precision provided by a single byte value, SSE instructions consider only unsigned 8-bit integers. To avoid negative values, bias calculation is performed based on the smallest value of the scoring matrix. This approach is described in Rognes [3] and Farrar [4]. Figure 3 describes the pseudocode of the ungapped extension procedure for two blocks of subsequences.

### Step 3: gapped extension

Ungapped alignments with significant similarities are passed in to this step to extend alignments with gap errors further. A significant amount of time can be spent on this activity, as shown by a BLASTP profiling study in [15], representing up to 30% of the total execution time. Parallelizing this step is also important to minimize overall execution time.

This is achieved as follows: the gapped extension is split into two sub-steps. In the first, *small *gapped extensions are considered. They are constrained by the number of permissible *ω *gaps with *λ *extensions to restrict the search space. The search space is also limited to a neighborhood of L amino acids on each side of the subset seed (L = 64). Again, if the score exceeds a threshold value, a full gapped extension (second sub-step) is computed using the NCBI-BLAST procedure. In this way, the results are similar to the BLAST output.

The reason for splitting this step into two stages is to make the computation more regular and, in this way, exhibit greater parallelism. The first part consists in computing many small gapped alignments where the search space is identical. The strategy is the same as the *banded Smith Waterman *algorithm strategy in WU-BLAST [16] with the *band length λ *and the *band width ω*. If the score of the left and right extensions exceeds a specified threshold *τ*_*sg*_, the second step using the full dynamic programming procedure is launched.

Small gapped extensions are also independent. SSE instructions may therefore be used again to compute a large number of them simultaneously. The ungapped alignments coming from step 2 are stored in a list. When this list contains at least K ungapped alignments, they are processed in SIMD mode.

**Figure 4:**
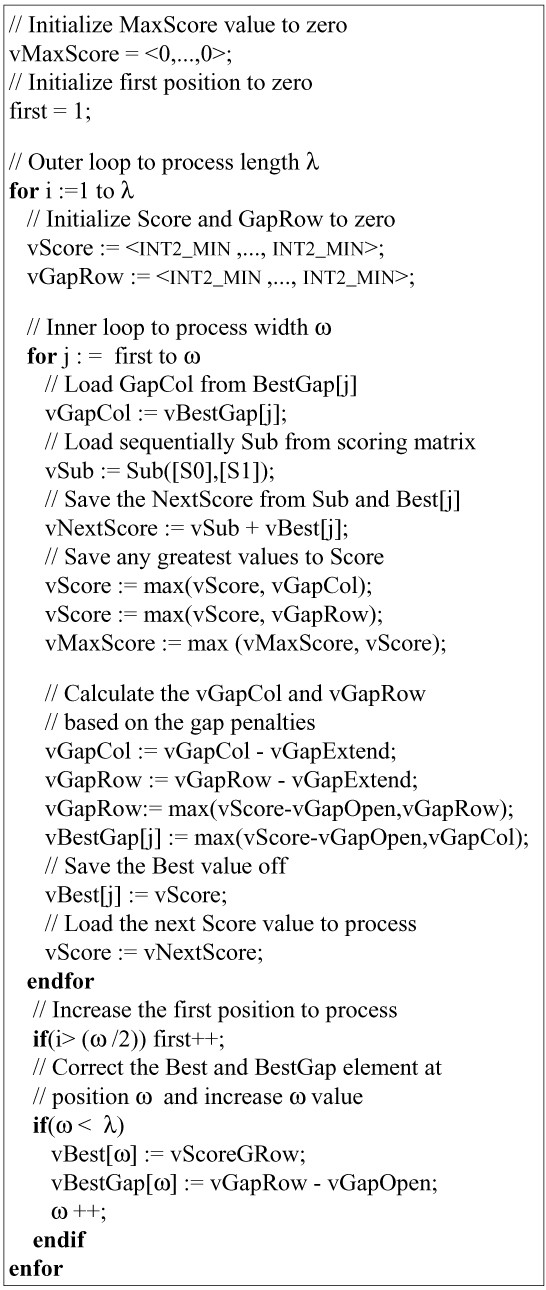
**pseudocode for *small gapped extension***. The pseudocode of small gapped extension procedure. Eight extensions are simultaneously processed and a score is stored on a 16-bit signed short integer.

Unlike ungapped extensions, however, pairs of subsequences are quite similar since a significant similarity has been detected during step 2. In addition, the length of the subsequences is longer (128 amino acids). Consequently, the score is unlikely to fit the range of an 8-bit integer. Thus, in this procedure, only 8 scores are computed in parallel, each score being stored in a 16-bit signed short integer. Figure 4 shows the pseudocode of the small gapped extension procedure.

An important point to be noted is that step 2 can generate many ungapped alignments belonging to the same final alignment, especially when strong similarities occur. In this case, several subset seeds are naturally included in the same alignment. With the approach discussed here, these subset seeds are systematically processed, even if they overlap, leading to high redundancy. To generate only one final alignment, a sorted list of all alignments already computed is stored in memory. Then, before launching a full gapped extension, a check is performed to see whether the small gap alignment to be extended is not included in the final alignment list. This list is common to all the extension threads.

### Statistical model

Like BLAST, PLAST uses Karlin-Altschul statistics [17,18] to evaluate the statistical significance of gapped alignments. An E-value is then associated to each alignment and is computed following the BLAST methodology. Since PLAST manages two banks, one is considered as a list of independent queries (-i option) and the other as the database (-d option). Compositions-based statistic [19] is also available for PLASTP and TPLASTN programs.

## Results and Discussion

This section presents the results of the experiments conducted on three versions of the PLAST algorithm for protein comparison: PLASTP, TPLASTN and PLASTX.

Sensitivity and selectivity were first evaluated using the receiver operating characteristic (*ROC*) and coverage versus errors per query (*EPQ*). Measurements show that results are comparable to BLAST (release 2.2.18). Execution time was then analyzed on standard multicore processors and also compared to BLAST. A speedup of 3 to 6 was achieved depending on the size and nature of the data.

### Receiver operating characteristic evaluation

First, the ROC statistical measure for PLASTP was computed using the method described in [20]. The data set was the SCOP database (release 1.73) with a maximum percentage identity of 40%, downloaded from the ASTRAL SCOP website [21,22]. This data set includes 7,678 sequences from 1,601 families. The 7,678 SCOP sequences are compared to the data set, and the results of all searches are pooled by E-value. True positives are query-subject pairs in the same family. Self-hits are ignored. For increasing E-value, the ROC score, for *n *false positives, is defined as:



T is the total number of true positives in the data set, i is the rank of the false positives, and t_*i *_is the number of true positives ranked ahead of the *i*th false positive.

**Figure 5:**
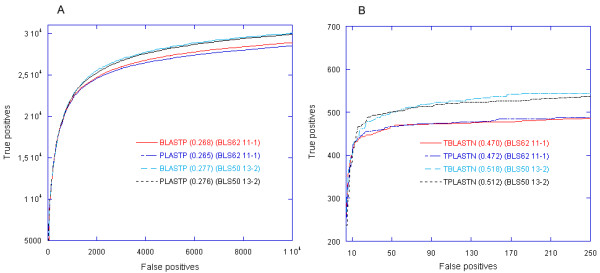
**ROC curve**. (A) The ROC curves for the SCOP/ASTRAL40 data set of PLASTP and BLASTP. (B) The ROC curves for the Yeast data set of TPLASTN and TBLASTN. The ROC_10000 _score in (A) and ROC_250 _score in (B) for each program are shown in parentheses after the program name.

The ROC curve was calculated for both PLASTP and BLASTP with the BLOSUM62 scoring matrix and gap penalty of 11-1 and with the BLOSUM50 scoring matrix and gap penalty of 13-2. Also, in both cases, the SEG filtering was disabled. The E-value was set to 10. The ROC curves of PLASTP and BLASTP are compared in Figure 5(A).

For computing the TPLASTN ROC curve, the data set was composed of the yeast (Saccharomyces cerevisiae) genome and a set of 102 proteins [23]. We used 102 proteins as queries against the yeast genome and, again, the results of all searches are pooled by E-value. All alignments were marked as true or false positives according to a careful human expert annotation [23]. Figure 5(B) shows the TPLASTN ROC curve.

As it can be seen, the PLAST and BLAST ROC curves are very close, but not identical. BLAST performs a little bit well than PLAST when its E-value is set to a high value. Actually, one of the main objectives of PLAST is to be included inside bioinformatics workflows to process large amount of data for automatic analysis, such as genome annotation. In that case, to increase confidence, the E-value is set to a much lower value. For example, setting the E-value to 10^-3 ^in the previous ROC analysis provides identical ROC curves between PLASTP and BLASTP (see Additional file 1).

### Coverage versus error plot

**Figure 6:**
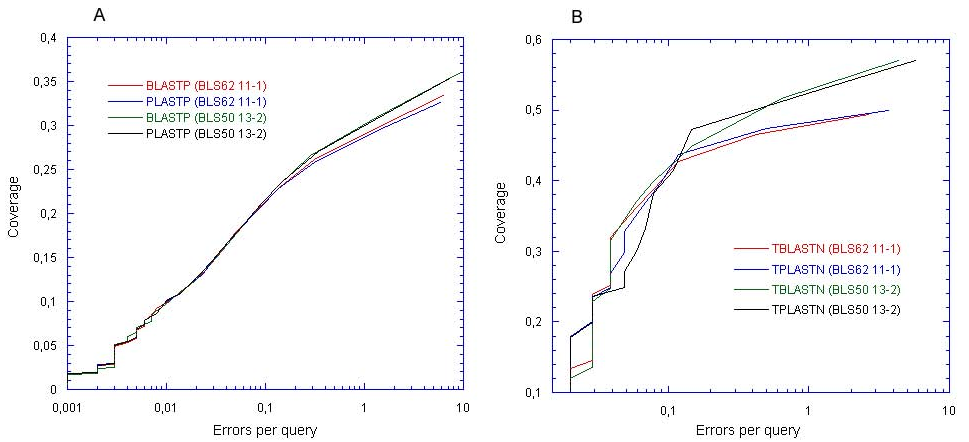
**Coverage versus error plot**. (A) The coverage versus error plots for the SCOP/ASTRAL40 data set of PLASTP and BLASTP. (B) The coverage versus error plots for the Yeast data set of TPLASTN and TBLASTN.

The coverage versus error plot was also used for evaluating the selectivity of PLAST. Instead of taking all alignments with a fixed E-value threshold, as in the ROC curve analysis, the E-value threshold was varied from 10^-50 ^to 10. Then for each threshold value, two parameters were measured: the coverage and errors per query (EPQ). The coverage is the number of true positives divided by the total number of true positives available in the data set. The EPQ is the number of false positives divided by the number of queries. The same two data sets were used for computing the coverage versus error plot for PLASTP, TPLASTN, BLASTP and TBLASTN. Figure 6 shows performance plots. Again, the plots obtained for the two program families, are very close.

### Execution time

In order to evaluate the ability of PLAST to manage large amounts of data, three data sets were made to test the PLASTP, TPLASTN and PLASTX programs, i.e. one data set for each program:

### Data set #1: PLASTP

• PROT-GB1-NR contains 2,977,744 protein sequences representing the first volume of the Genbank nonredundant protein database (1,000 Mega aa);

• PROT-SCOP-1K, PROT-SCOP-3K and PROT-SCOP-10K contain respectively 1,000 protein sequences (0.185 Mega aa), 3,000 protein sequences (0.434 Mega aa) and 10,000 protein sequences (1.871 Mega aa) selected from the SCOP database.

### Data set #2: TPLASTN

• DNA-HUMAN-CHR1 is human chromosome 1 (NCBI Mar. 2008, 220 Mega nt);

• PROT-GB-1K, PROT-GB-3K and PROT-GB-10K contain respectively 1,000 protein sequences (0.336 Mega aa), 3,000 protein sequences (1.025 Mega aa) and 10,000 protein sequences (3.433 Mega aa) selected from the Genbank nonredundant protein database.

### Data set #3: PLASTX

• SWPROT is UniProtKB/Swiss-Protis (Release 56.2, 398,181 protein sequences, 144 Mega aa);

• DNA-GB-1K, DNA-GB-3K and DNA-GB-10K contain respectively 1,000 DNA sequences (1.031 Mega nt), 3,000 DNA sequences (3.172 Mega nt) and 10,000 DNA sequences (10.175 Mega nt) selected from the gbvrl Genbank division.

The hardware platform is a 2.6 GHz Xeon Core 2 Quad processor with 8 GB of RAM running Linux Fedora 7. This platform is thus able to run 8 threads in parallel. The Xeon Core 2 processor has a standard SSE instruction set.

### Comparison with BLAST

Each PLAST program was run with its specific fiata set. For the purpose of comparison, the BLASTP, TBLASTN and BLASTX programs (release 2.2.18) were also run with the same data set, with the multithreading option enabled (-a option). **blastall **was run as follows:

blastall -p program of BLAST -m 8 -a number of threads -e E-value

Experiments were performed on three runs:

• # threads = 2, E-value = 10^-3 ^(Table 1)

**Table 1:** Multicore with 2 threads and E-value equal to 10^-3^

	**protein vs protein**			**protein vs DNA**			**DNA vs protein**		
	
**query bank**	**BLASTP**	**PLASTP**	**speedup**	**TBLASTN**	**TPLASTN**	**speedup**	**BLASTX**	**PLASTX**	**speedup**
1K	4380	1446	3.02	805	319	2.52	2261	554	4.08
3K	10860	2602	4.17	2344	556	4.21	6989	1591	4.39
10K	52131	12415	4.19	7971	1416	5.26	21667	4981	4.34

Comparison of performance of BLAST and PLAST families running with 2 threads. The E-value cutoff is set to 10^-3 ^and option "-m8" of BLAST is enabled. BLAST is run in multithread mode (-a 2). Execution times are given in seconds.

• # threads = 8, E-value = 10^-3 ^(Table 2)

**Table 2:** Multicore with 8 threads and E-value equal to 10^-3^

	**protein vs protein**			**protein vs DNA**			**DNA vs protein**		
	
**query bank**	**BLASTP**	**PLASTP**	**speedup**	**TBLASTN**	**TPLASTN**	**speedup**	**BLASTX**	**PLASTX**	**speedup**
1K	1530	506	3.02	384	117	3.28	651	174	3.74
3K	4206	898	4.46	1068	186	5.74	1999	451	4.43
10K	21450	3807	5.60	3659	428	8.54	6237	1418	4.39

Comparison of performance of BLAST and PLAST families running with 8 threads. The E-value cutoff is set to 10^-3 ^and option "-m8" of BLAST is enabled. BLAST is run in multithread mode (-a 8). Execution times are given in seconds.

• # threads = 2, E-value = 10 (Table 3)

**Table 3:** Multicore with 2 threads and E-value equal to 10

	**protein vs protein**			**protein vs DNA**			**DNA vs protein**		
	
**query bank**	**BLASTP**	**PLASTP**	**speedup**	**TBLASTN**	**TPLASTN**	**speedup**	**BLASTX**	**PLASTX**	**speedup**
1K	4836	1521	3.17	1003	360	2.78	2286	558	4.08
3K	12298	2861	4.29	2881	632	4.55	7010	1631	4.29
10K	58145	14004	4.15	9480	1631	5.81	21774	5002	4.35

Comparison of performance of BLAST and PLAST families running with 2 threads. The E-value cutoff is set to 10 and option "-m8" of BLAST is enabled. BLAST is run in multithread mode (-a 2). Execution times are given in seconds.

In all cases, the BLOSUM62 matrix was used with gap-open penalty and gap-extension penalty set respectively to 11 and 1 (default BLAST parameters). Tables 1 to 3 show the time spent (in seconds) for each run and the speedup of PLAST compared to BLAST.

An E-value of 10^-3 ^is a reasonable value when performing intensive sequence comparison. However, setting the E-value to 10 had no significant impact on the execution time.

It can be seen that for each experiment, significant speedup is obtained compared to BLAST. More precisely, the speedup obtained (each measure was performed with an identical number of threads) increased with the size of the data set.

To evaluate PLAST sensitivity for large databases, sets of alignments reported by PLAST and BLAST were compared using two large sets of data: GB1-NR versus PROT-SCOP-1K (PLASTP) and SWPROT versus DNA-GB-1K (PLASTX). Two alignments are considered equivalent if they overlap by more than 70%. An alignment A is included in an alignment B if alignment A belongs to alignment B. A misalignment occurs if an alignment found by one program is not found by the other. The E-value threshold was varied from 10 to 10^-3^. For each threshold value, the misalignments of PLAST and BLAST were calculated for the two data sets as follows:

• PLAST_*miss *_= BLAST_*total *_- BLAST_*include *_- Identical

• BLAST_*miss *_= PLAST_*total *_- PLAST_*include *_- Identical

where BLAST_*total *_and PLAST_*total *_are the numbers of alignments found respectively by BLAST and PLAST; BLAST_*include *_and PLAST_*include *_are the numbers of alignments included respectively in PLAST_*total *_and BLAST_*total *_of BLAST and PLAST; Identical is the number of equivalent alignments between BLAST and PLAST. The results are shown in Tables 4 and 5. See Additional file 2 for results on the 3K and 10K data sets.

**Table 4:** Misalignments of PLASTP and BLASTP

**E-value**	**10**	**1**	**10**^**-1**^	**10**^**-2**^	**10**^**-3**^
BLASTP_*total*_	556570	507225	462673	423919	394887
PLASTP_*total*_	537892	497933	464238	422466	394636
Identical	513096	477982	442854	409437	383746
BLASTP_*include*_	11227	9135	7586	6181	5259
PLASTP_*include*_	3880	2868	2277	1570	1250
BLASTP_*miss*_	20916 (3.9%)	17083 (3.4%)	19271 (4.1%)	9640 (2.2%)	10890 (2.7%)
PLASTP_*miss*_	32247 (5.9%)	20108 (4.0%)	12232 (2.6%)	8301 (1.9%)	5882 (1.4%)

Misalignments of PLASTP and BLASTP for GB1-NR versus PROT-SCOP-1K for different E-values.

**Table 5:** Misalignments of PLASTX and BLASTX

**E-value**	**10**	**1**	**10**^**-1**^	**10**^**-2**^	**10**^**-3**^
BLASTX_*total*_	127124	104474	96559	91760	88127
PLASTX_*total*_	123425	101789	96051	90736	87085
Identical	113336	98660	93267	89285	85982
BLASTX_*include*_	1694	1240	972	794	655
PLASTX_*include*_	1317	823	591	398	268
BLASTX_*miss*_	8772 (7.5%)	2306 (2.2%)	2193 (2.2%)	1053 (1.0%)	835 (0.9%)
PLASTX_*miss*_	12094 (9.5%)	4574 (4.3%)	2701 (2.8%)	1681 (1.8%)	1490 (1.6%)

Misalignments of PLASTX and BLASTX for SWPROT versus DNA-GB-1K for different E-values.

The two programs do not find exactly the same alignments. This is due to the difference between the heuristics used to discover the seeds. Nonetheless, for the small E-values generally encountered when using PLAST, the results are very close.

### PLAST performance analysis

Table 6 shows the execution time (in seconds) of the three PLAST programs relative to the number of threads and data sets. A first point is that performance increases with the size the data set, whatever the number of threads. This is mainly due to the architecture of the algorithm, which presents great computational locality, especially in step 2 (ungapped extension). This locality favors the use of the memory cache system and minimizes external memory access, which is a slow process compared to the processor internal clock frequency.

**Table 6:** Execution time of the three PLAST programs

**program**	**PLASTP**			**TPLASTN**			**PLASTX**		
query bank	1K	3K	10K	1K	3K	10K	1K	3K	10K
1 thread	2704	5024	24374	570	1053	2810	1081	3090	9730
2 threads	1446	2602	12415	319	556	1416	554	1591	4981
4 threads	847	1480	7370	188	310	773	303	842	2620
8 threads	506	898	3807	117	186	428	174	451	1418

Performance of the three PLAST programs running with multithreading mode. The E-value cutoff is set to 10^-3^. Execution times are given in seconds.

**Figure 7:**
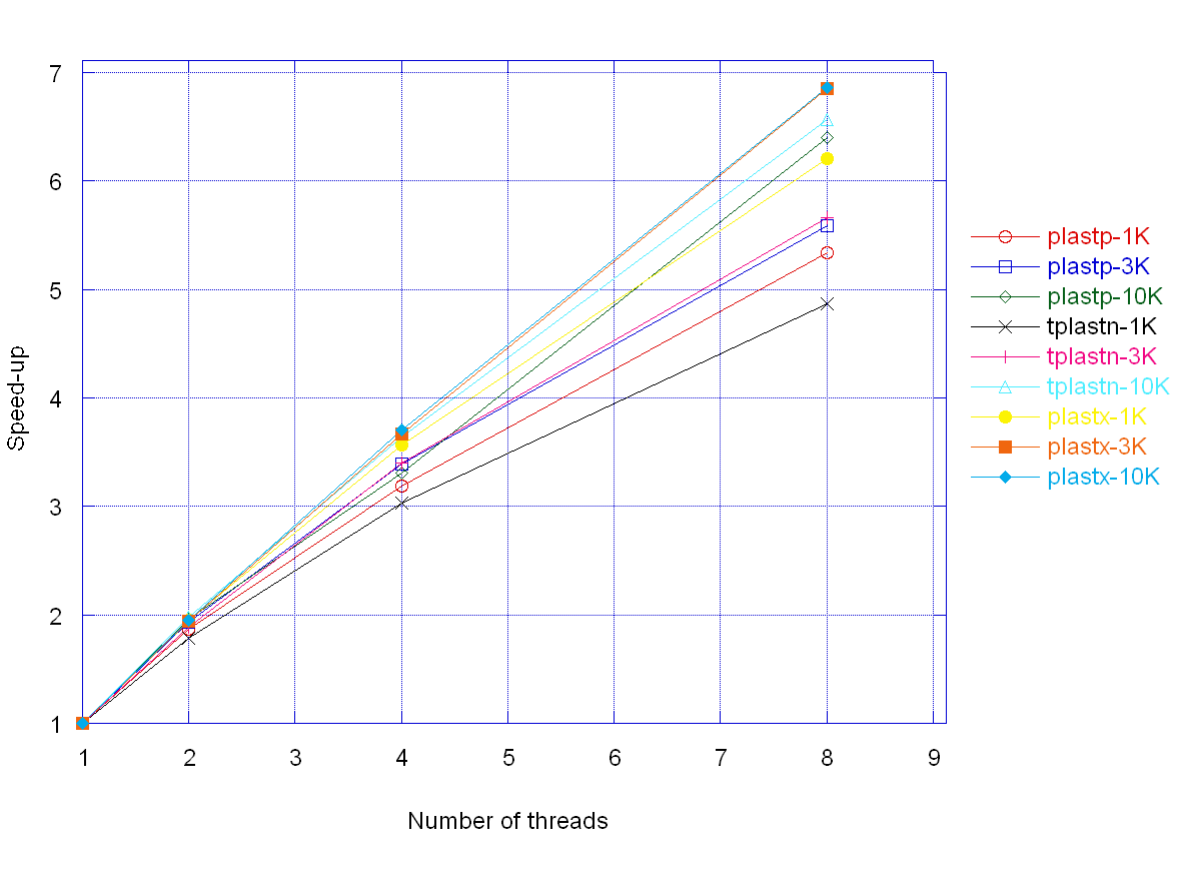
**Speedup of the three PLAST programs**. Speedup of the three PLAST programs relative to the number of threads and data sets. The E-value cutoff is set to 10^-3^.

A second point is the scalability of the PLAST algorithm when the number of threads increases. Figure 7 depicts speedup as a function of the number of threads. It clearly highlights limitations due to the sequential indexing part of the program as explained by Table 7, which shows the time required for step 1 as a percentage of overall execution time. As stated by Amdahl's law [24], the speedup of a program using multiple processors is limited by the time required for the sequential fraction (P) of the program. The maximum speedup is bounded by 1/(1 - *P*). Here, even if the indexing part represents a small fraction of the execution time, it represents a serious obstacle for the next generation of microprocessors, which will include a great number of cores on the same die.

**Table 7:** Percentage of indexing time overall in the three PLAST programs

**program**	**PLASTP**			**TPLASTN**			**PLASTX**		
query bank	1K	3K	10K	1K	3K	10K	1K	3K	10K
Indexing time (1 thread)	3.0%	1.6%	0.3%	6.5%	3.5%	1.4%	1.2%	0.4%	0.1%
Indexing time (8 threads)	15.8%	8.9%	2.1%	31.6%	19.9%	8.9%	7.5%	2.9%	1.0%

Percentage of indexing time overall in the three PLAST programs with number of threads equal to 1 and 8.

**Figure 8:**
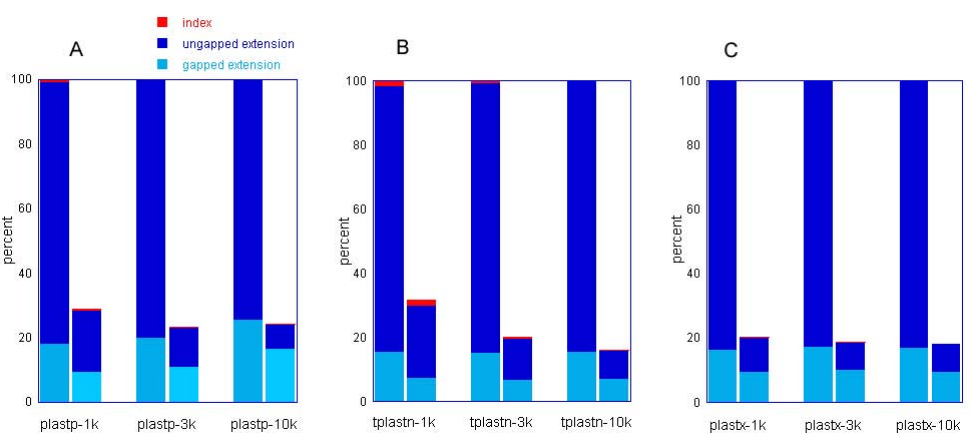
**PLAST profile**. The profiles of the three PLAST programs, with and without SSE instructions. Each PLAST program was run with its specific data set: (A) PLASTP; (B) TPLASTN; (C) PLASTX.

To measure the benefit of the SSE accelerations, profiling was performed, as shown in Figure 8. The same data set was used. The reference (100%) was the execution time without the use of the SSE instructions. More details can be found in Additional file 3 to compare single-thread and non-SSE execution time between BLAST and PLAST.

It can be seen that the ungapped extension represents a high percentage of computation time and that it can be considerably reduced with the SSE instructions. SSE instructions have a more modest impact on gap extensions.

## Conclusion

PLAST primarily focuses on intensive sequence comparison applications, unlike BLAST, which is well optimized for scanning large genomic databases. It has been designed to manage large amounts of data and provides the best performance for such applications.

PLAST is faster than BLAST, while providing comparable sensitivity with the same Karlin-Altschul statistics model. Results are not strictly identical since the heuristics for detecting alignments are different, even if both are based on seed techniques. PLAST integrates a 4-character subset seed approach while BLAST starts an extension when two 3-character seeds are located in a close neighborhood.

BLAST and PLAST do not exactly target the same bioinformatics applications, even if PLAST aims to produce identical results. BLAST performs fast and sensitive scans of genomic databases. To detect low similarities, the user can set a high E-value and then analyse and interpret alignments. In that case, BLAST is better suited than PLAST since sensitivity is a little bit better.

On the other hand, PLAST performs fast bank to bank comparison and results are expected to be piped to further automatic analysis. In this context, the E-value is generally set to a much lower value, leading PLAST to produce similar results compared to BLAST.

PLAST has been designed to target the current and next generations of microprocessors that are - and will remain - parallel machines. Two types of parallelism are taken into consideration: multithreading (targeting multi- and manycore architectures) and SIMD (use of SSE instructions). These two modes of parallelism are combined to obtain maximum performance from the architecture of current and future microprocessors. For instance, the next generation of the new Intel set of SSE instructions, called AVX [25], which extends the SIMD integer registers to 256 bits and 512 bits, will be directly operational through the PLAST implementation. Similarly, advanced micro architectures, like the Intel Larrabee project [26] or the China Goldson-T manycore project [27], prefigure tomorrow's parallel hardware platforms, where PLAST parallelism will be fully exploited.

Since bank indexing is done on-the-fly, PLAST requires no preformatting processes (such as formatdb) before it can be run. The two banks simply need to be in the widely used FASTA format. On the other hand, PLAST does not print alignments in the default BLAST output format. The main reason for this is that PLAST is not intended for interactive use, but rather as a building block in the primary stages of computational workflows for more advanced bioinformatics studies. Hence, the default PLAST output corresponds to the "-m 8" BLAST option, which simply summarizes the features of all alignments. This format is comprehensive for humans and very easy to handle for computers.

PLAST is a 3-step algorithm where the two most time-consuming steps have been parallelized. On an 8-core architecture, corresponding to a current medium-range platform, good speedup is achieved. For larger configurations with 16 or 32 cores, speedup will be limited by the indexing part which, in the current implementation, is a purely sequential part. The next PLAST challenge is to parallelize this step.

The PLAST family programs are currently focusing on protein sequences. PLASTN is not yet included in the current package. Work is still in progress to achieve an efficient version that takes into account the specifics of DNA sequences, especially for the ungapped step extension.

## Availability and requirements

Project name: PLAST

Project home page: http://www.irisa.fr/symbiose/projects/plast

Operating system(s): Linux

Programming language: C

License: CECILL

Restrictions for use by non-academics: none.

## Authors' contributions

Both authors contributed to the design of the PLAST algorithm. The C implementation was mostly done by VHN. All authors read and approved the final manuscript.

## Supplementary Material

Additional file 1 **Supplementary ROC curve**. The ROC curves for the SCOP/ASTRAL40 data set of PLASTP and BLASTP with E-value of 10^-3 ^and ROC curves for the Yeast data set of TPLASTN and TBLASTN with E-value of 1. Click here for file

Additional file 2 **Misalignments of PLAST and BLAST**. The sensitivity results of BLAST and PLAST for four large sets of data: GB1-NR versus PROT-SCOP-3K, GB1-NR versus PROT-SCOP-10K, SWPROT versus DNA-GB-3K and SWPROT versus DNA-GB-10K. Click here for file

Additional file 3 **Single-threaded performance**. The comparison of performance of BLAST and PLAST families running with single-threaded and non-SSE. Click here for file
