# Supplementary material for: PLAST: parallel local alignment search tool for database comparison
Source: BMC Bioinformatics. 2009 Oct 12;10:329. doi: 10.1186/1471-2105-10-329 (PMC2770072; doi:10.1186/1471-2105-10-329)
Supplement: Additional file 1 — Supplementary ROC curve. The ROC curves for the SCOP/ASTRAL40 data set of PLASTP and BLASTP with E-value of 10-3 and ROC curves for the Yeast data set of TPLASTN and TBLASTN with E-value of 1. [file 1471-2105-10-329-S1.PDF]

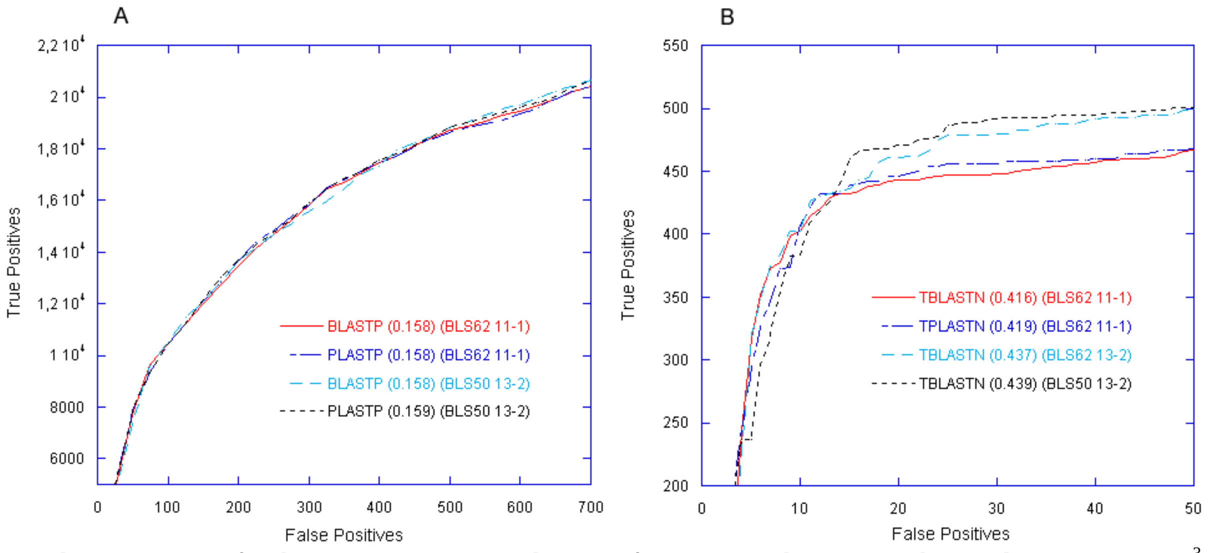

(A) The ROC curves for the SCOP/ASTRAL40 data set of PLASTP and BLASTP, the E-value was set to 10^{-3}. (B) The ROC curves for the Yeast data set of TPLASTN and TBLASTN, the E-value was set to 1. The ROC_{700} score in (A) and ROC_{50} score in (B) for each program are shown in parentheses after the program name.
