# Supplementary material for: PLAST: parallel local alignment search tool for database comparison
Source: BMC Bioinformatics. 2009 Oct 12;10:329. doi: 10.1186/1471-2105-10-329 (PMC2770072; doi:10.1186/1471-2105-10-329)
Supplement: Additional file 2 — Misalignments of PLAST and BLAST. The sensitivity results of BLAST and PLAST for four large sets of data: GB1-NR versus PROT-SCOP-3K, GB1-NR versus PROT-SCOP-10K, SWPROT versus DNA-GB-3K and SWPROT versus DNA-GB-10K. [file 1471-2105-10-329-S2.PDF]

Evalue	10	1	10^{-1}	10^{-2}	10^{-3}
$BLASTP_{total}$	2,265,188	1,957,969	1,681,873	1,458,895	1,311,728
$PLASTP_{total}$	2,134,996	1,891,731	1,682,760	1,444,123	1,304,392
Identical	2,042,719	1,821,964	1,599,772	1,403,789	1,270,949
$BLASTP_{include}$	42,583	34,500	27,492	22,156	18,369
$PLASTP_{include}$	9,674	7,009	5,467	3,787	2,990
$BLASTP_{miss}$	82,603 (3.8%)	62,758 (3.3%)	77,521 (4.6%)	36,547 (2.5%)	30,453 (2.3%)
$PLASTP_{miss}$	179,886 (7.9%)	101,505 (5.1%)	54,609 (3.2%)	32,950 (2.2%)	22,410 (1.8%)

Table 1: Misalignments of PLASTP and BLASTP for GB1-NR versus PROT-SCOP-3K for different E-values

Evalue	10	1	10^{-1}	10^{-2}	10^{-3}
$BLASTP_{total}$	11,614,806	10,550,719	9,574,744	8,747,939	8,144,404
$PLASTP_{total}$	11,333,685	10,434,198	9,659,926	8,744,933	8,162,232
Identical	10,838,876	10,037,458	9,231,407	8,492,530	7,944,855
$BLASTP_{include}$	217,981	180,888	148,387	120,024	100,852
$PLASTP_{include}$	65,476	51,777	42,181	31,738	25,901
$BLASTP_{miss}$	429,333 (3.8%)	344,963 (3.3%)	386,338 (3.9%)	220,665 (2.5%)	191,476 (2.3%)
$PLASTP_{miss}$	557,949 (4.8%)	332,373 (3.1%)	194,950 (2.0%)	135,385 (1.5%)	98,697 (1.3%)

Table 2: Misalignments of PLASTP and BLASTP for GB1-NR versus PROT-SCOP-10K for different E-values

Evalue	10	1	10^{-1}	10^{-2}	10^{-3}
$BLASTX_{total}$	371,880	306,345	282,946	268,854	258,804
$PLASTX_{total}$	363,491	299,675	282,350	266,740	257,147
Identical	331,268	289,037	273,265	261,872	253,115
$BLASTX_{included}$	5,055	3,682	2,832	2,211	1,970
$PLASTX_{included}$	4,491	2,829	2,034	1,355	1,067
$BLASTX_{miss}$	27,732 (7.6%)	7,809 (2.6%)	7,051 (2.5%)	3,513 (1.3%)	2,965 (1.1%)
$PLASTX_{miss}$	35,557 (9.5%)	13,626 (4.4%)	6,849 (2.4%)	4,771 (1.7%)	3,719 (1.4%)

Table 3: Misalignments of PLASTX and BLASTX for SWPROT versus DNA-GB-3K for different E-values

Evalue	10	1	10^{-1}	10^{-2}	10^{-3}
$BLASTX_{total}$	1,236,542	1,017,329	940,507	893,046	856,538
$PLASTX_{total}$	1,205,960	987,950	932,160	881,113	847,576
Identical	1,100,825	956,029	904,680	866,478	835,016
$BLASTX_{include}$	22,292	16,728	13,202	10,497	8,735
$PLASTX_{include}$	14,581	9,215	6,693	4,477	3,306
$BLASTX_{miss}$	90,554 (7.5%)	22,706 (2.2%)	20,787 (2.2%)	10,158 (1.0%)	9,254 (1.1%)
$PLASTX_{miss}$	113,425 (9.1%)	44,572 (4.3%)	22,625 (2.4%)	16,071 (1.8%)	12,787 (1.5%)

Table 4: Misalignments of PLASTX and BLASTX for SWPROT versus DNA-GB-10K for different E-values
