# Supplementary material for: PLAST: parallel local alignment search tool for database comparison
Source: BMC Bioinformatics. 2009 Oct 12;10:329. doi: 10.1186/1471-2105-10-329 (PMC2770072; doi:10.1186/1471-2105-10-329)
Supplement: Additional file 3 — Single-threaded performance. The comparison of performance of BLAST and PLAST families running with single-threaded and non-SSE. [file 1471-2105-10-329-S3.PDF]

	protein vs protein			protein vs DNA			DNA vs protein		
query bank	BLASTP	PLASTP	speedup	TBLASTN	TPLASTN	speedup	BLASTX	PLASTX	speedup
1K	8,151	2,704	3.01	1,444	573	2.53	4,458	1,074	4.15
3K	19,910	5,024	3.96	4,256	1,053	4.04	13,716	3,085	4.44
10K	92,973	24,359	3.81	14,380	2,810	5.11	42,569	9,710	4.38

Table 1: Comparison of performance of BLAST and PLAST families running with single-threaded. The E-value cutoff is set to 10^{-3} and option "-m8" of BLAST is enabled. Execution times are given in seconds.

	protein vs protein			protein vs DNA			DNA vs protein		
query bank	BLASTP	PLASTP*	speedup	TBLASTN	TPLASTN*	speedup	BLASTX	PLASTX*	speedup
1K	8,151	9,737	0.84	1,444	1,904	0.76	4,458	5,553	0.80
3K	19,910	22,161	0.90	4,256	5,502	0.77	13,716	17,424	0.79
10K	92,973	104,124	0.89	14,380	18,323	0.78	42,569	56,055	0.76

Table 2: Comparison of performance of BLAST and PLAST (**without SSE instructions**) families running with single-threaded. The E-value cutoff is set to 10^{-3} and option "-m8" of BLAST is enabled. Execution times are given in seconds. (*) represents PLAST without SSE instructions. It's clear that speedup is bought by SSE instructions and that the structure of the algorithm is interesting only if such instructions are available.
